# Structure and function of FusB: an elongation factor G-binding fusidic acid resistance protein active in ribosomal translocation and recycling

**DOI:** 10.1098/rsob.120016

**Published:** 2012-03

**Authors:** Xiaohu Guo, Kristin Peisker, Kristina Bäckbro, Yang Chen, Ravi Kiran Koripella, Chandra Sekhar Mandava, Suparna Sanyal, Maria Selmer

**Affiliations:** Department of Cell and Molecular Biology, BMC, PO Box 596, SE 751 24, Uppsala, Sweden

**Keywords:** FusB, elongation factor G, fusidic acid, antibiotic resistance

## Abstract

Fusidic acid (FA) is a bacteriostatic antibiotic that locks elongation factor G (EF-G) to the ribosome after GTP hydrolysis during elongation and ribosome recycling. The plasmid pUB101-encoded protein FusB causes FA resistance in clinical isolates of *Staphylococcus aureus* through an interaction with EF-G. Here, we report 1.6 and 2.3 Å crystal structures of FusB. We show that FusB is a two-domain protein lacking homology to known structures, where the N-terminal domain is a four-helix bundle and the C-terminal domain has an alpha/beta fold containing a C4 treble clef zinc finger motif and two loop regions with conserved basic residues. Using hybrid constructs between *S. aureus* EF-G that binds to FusB and *Escherichia coli* EF-G that does not, we show that the sequence determinants for FusB recognition reside in domain IV and involve the C-terminal helix of *S. aureus* EF-G. Further, using kinetic assays in a reconstituted translation system, we demonstrate that FusB can rescue FA inhibition of tRNA translocation as well as ribosome recycling. We propose that FusB rescues *S. aureus* from FA inhibition by preventing formation or facilitating dissociation of the FA-locked EF-G–ribosome complex.

## 2. Introduction

Fusidic acid (FA) is a bacteriostatic antibiotic that was first isolated from the fungus *Fusidium coccineum* in the early 1960s [1]. FA blocks bacterial protein synthesis by locking elongation factor G (EF-G) to the ribosome [2]. Clinically, FA is mainly used against staphylococcal infections, often in combination with other drugs to prevent resistance development.

EF-G is a translational GTPase catalysing two different steps of protein synthesis (reviewed by Schmeing & Ramakrishnan [3]). First, EF-G is needed for translocation of tRNAs and mRNA with respect to the ribosomal 30S subunit to make a new mRNA codon available for decoding. Second, EF-G acts together with ribosome recycling factor (RRF) in splitting of the ribosomal post-termination complex. In both of these steps, GTP hydrolysis by EF-G is used as an energy source, and in both cases FA prevents the release of EF-G from the ribosome after GTP hydrolysis [2,4]. Since FA locks EF-G in a defined state with GDP on the ribosome, the drug has also been used as a tool in structural studies of ribosome–EF-G complexes by cryo-electron microscopy and crystallography [5–7]. These structures display EF-G conformations that are similar to what is observed in a complex blocked with a non-hydrolysable GTP analogue [8]. In contrast, the observed EF-G conformations are distinctly different from isolated crystal structures of EF-G in apo form [9,10] with GDP [11,12] or with a GTP analogue [13]. Most of these structures are of *Thermus thermophilus* EF-G and are in similar global conformations, probably owing to crystal packing. The main conformational change in EF-G occurs between two blocks of the structure, consisting of domains I–II and domains III–V, and is triggered by a combination of ribosome interactions and conformational changes of the two switch regions upon GTP hydrolysis.

The FA binding site was for the first time visualized in the FA-locked 3.6 Å crystal structure of EF-G with the 70S ribosome [6]. FA binds at the interface between domains I, II and III of EF-G, and only displays high affinity to the ribosome-bound EF-G. The structure clarified that the drug locks EF-G in a conformation between the ribosome-binding GTP state and the dissociating GDP state [6]. Specifically, FA binds to EF-G after GTP hydrolysis, when the switch I region has left its ordered GTP conformation and prevents switch II from leaving its GTP-like conformation. Thereby, the drug stops the conformational change of EF-G that is presumably needed for dissociation from the ribosome. A recent study shows that the recycling step *in vitro* is inhibited at more than 1000-fold lower FA concentration than the translocation reaction [14]. However, it remains unknown whether either or both of these steps are the natural targets of FA *in vivo*.

It was recognized early that FA resistance could reside in EF-G [15]. To date, the identified types of FA resistance are defined as *fusA*, *fusB*, *fusC*, *fusD* and *fusE* (reviewed by Farrell *et al*. [16]). Mutations in the drug target, EF-G, belong to the *fusA* class [17,18]. While some of these directly affect the FA-binding site [6], others perturb EF-G–ribosome contacts, conformational dynamics of EF-G or the stability of EF-G domains [10,12,19], reflecting that FA only binds to a defined conformation of EF-G on the ribosome. The *fusE* mutants have frameshift or truncation mutations in the *rplF* gene encoding ribosomal protein L6 [18]. These *in vitro*-selected mutants are the only known ribosomal FA resistance mutations and affect a contact area with EF-G [6,10].

Plasmid-based resistance towards FA in *S. aureus* was first demonstrated nearly four decades ago [20,21], but it was only more recently that the resistance-causing gene *fusB* was identified on the 22 kB pUB101 plasmid [22,23]. FusB is a 25 kD protein that can provide low-level FA resistance in *S. aureus* [22,23]. It displays sequence homology to a *Listeria monocytogenes* fibronectin-binding protein [22,23] implicated in host-cell attachment [24], but does not bind to fibronectin [23]. FusB does not display sequence homology to any protein of known three-dimensional structure and its evolutionary origin remains to be analysed. The chromosomally encoded FusB homologue FusC exists in some *S. aureus* strains [25], and FusD has been found to cause the inherent resistance of *Staphylococcus saprophyticus* [25]. Recent studies indicate that *fusB* and *fusC* are the most common types of FA resistance in recent clinical isolates of methicillin-sensitive *S. aureus*, while *fusA* is more common in methicillin-resistant *S. aureus* [25,26].

In pull-down experiments, His-tagged FusB pulled out one single protein, identified as EF-G, from *S. aureus* cell extract, while no protein was pulled out from *E. coli* extract. Since FusB could protect an *S. aureus*-based *in vitro* translation system from FA inhibition, but failed to do the same to an *E. coli*-based system, it was concluded that the interaction between FusB and EF-G is crucial for the FusB resistance mechanism [23].

Beyond that, the mechanism of action of FusB is unknown. In this study, we have solved the crystal structure of FusB, mapped its binding site on EF-G and demonstrated that FusB can rescue FA inhibition in elongation as well as in recycling. We conclude that FusB provides FA resistance by preventing formation or facilitating dissociation of the FA-locked EF-G–ribosome complex in both of these steps.

## 3. Results

### 3.1. Structure determination of FusB

An N-terminally His-tagged version of FusB was cloned from plasmid pUB101 present in clinical isolates of *S. aureus* [21] and overexpressed in *E. coli*. FusB was crystallized under several different conditions at the high-throughput crystallization facility in Grenoble, France. After optimization, crystals in space group *P*2_1_2_1_2 diffracting to 1.6 Å resolution grew in polyethylene glycol at pH 5.5, and crystals in space group *P*1 diffracting to 2.3 Å resolution grew in polyethylene glycol at pH 8.1. The structure was solved using single-wavelength anomalous dispersion with *P*2_1_2_1_2 crystals soaked in sodium iodide and refined against the native data. The *P*1 crystal structure was solved by molecular replacement using the refined *P*2_1_2_1_2 FusB structure as search model. FusB crystallized as a dimer in the asymmetric unit of both crystal forms. The A molecule of the *P*2_1_2_1_2 structure was fully ordered apart from the His-tag, whereas the other molecules displayed disorder in one or several loop regions. In the *P*2_1_2_1_2 structure, we observed a continuous density across the domain interface between residues Ser15, Lys162 and Thr200 that we failed to interpret (electronic supplementary material, figure S1). Attempts to identify any bound ligand by mass spectrometry failed. Below, unless stated otherwise, the higher-resolution *P*2_1_2_1_2 structure will be described.

### 3.2. Overall structure

**Figure 1.**
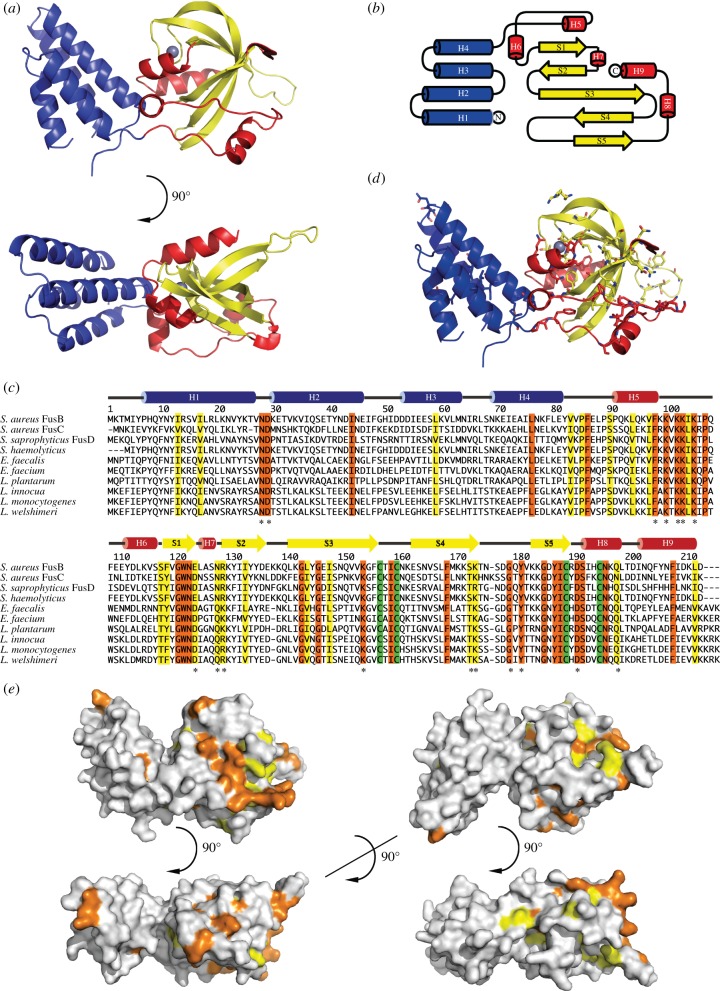
Overall structure of FusB. (*a*) Cartoon diagram of the FusB structure. Domain I is shown in blue and domain II in yellow and red. (*b*) Topology of FusB. Colours as in (*a*). (*c*) Sequence alignment of FusB with homologues having experimental evidence for FA resistance (reference after each accession code). *S. aureus* FusB: NP_932197.1 [22,23], *S. aureus* FusC: YP_042173.1 [25], *S. saprophyticus* FusD: YP_302255.1 [25], *S. haemolyticus* FusB: CAJ43426.1 [27], *E. faecalis* T11 fibronectin-binding protein: ZP_05595118.1 [28], *E. faecium* D344SRF fibronectin-binding protein: ZP_06447277.1 [28]*, L. plantarum* fibronectin-binding protein: CCC77620.1 [29], *L. innocua* putative fibronectin-binding protein: NP_470072.1 [23], *L. monocytogenes* str. 1/2a F6854 fibronectin-binding protein: ZP_00232898.1 [30], *L. welshimeri* fibronectin-binding protein: YP_848891.1 [30]. The secondary structure is indicated on top, conserved residues are marked in orange, conservatively substituted residues in yellow and conserved zinc ligands in green. Stars indicate surface-exposed conservations. (*d*) Location of conserved residues in the FusB structure. Conserved and conservatively substituted residues are shown as sticks. (*e*) Sequence conservation mapped on the FusB surface. Conserved residues are shown in orange and conservatively substituted residues in yellow. The four views are 90° apart and the first two correspond to the ones in (*a*).

The FusB structure consists of two domains, together forming a structure of approximately 70 × 37 × 30 Å size. The N-terminal domain is an elongated up–down four-helix bundle, while the C-terminal domain is a more spherical alpha/beta domain stabilized by a zinc ion (figure 1*a,b*).

### 3.3. Sequence analysis of *Staphylococcus aureus* FusB

*Staphylococcus aureus* FusB displayed significant sequence homology to about 170 other protein sequences, the majority from bacilli and enterococci, found in a BLAST search (data not shown). Out of these, we chose to align the *S. aureus* FusB sequence to a subset of nine sequences with experimental evidence for a role in FA resistance or for inherent FA resistance of the respective species (figure 1*c*; references in figure legend). The *Staphylococcus haemolyticus* protein is a truncated version of FusB, and the other sequences display 30 to 46 per cent sequence identity to FusB (the *Lactobacillus plantarum* protein being least similar). The resulting sequence alignment allowed mapping of the conserved residues on the FusB structure (figure 1*d*,*e*). In the N-terminal domain, most of the conserved residues are part of the hydrophobic core, and only two surface-exposed residues Asn28 and Asp29 at the tip of the domain are conserved. In contrast, the C-terminal domain, as discussed below, contains several patches with exposed conserved residues.

### 3.4. C-terminal domain

The C-terminal domain as a whole has a novel fold that is not present in any other structure in the protein data bank (PDB), assessed using the Dali and VAST servers. After an inter-domain linker, the domain starts with a short helix H5 and a long loop. The helix and the loop contain seven lysine residues from amino acids 93 to 105. Four of these—Lys99, Lys101, Lys102 and Lys104—are strictly conserved in the aligned FusB homologues (figure 1*c*,*d*). All the lysines in the helix and the loop are pointing outwards from the protein, repelling each other. The B-factors of the loop are above average, but the hydrophobic residues of the loop (Phe98, Val101, Ile104) participate in the hydrophobic core and keep the backbone conformation similar in all four FusB molecules.

After another short helix, the domain continues in five-stranded anti-parallel beta sheet, forming a jaw-like structure biting into a V-shape of two short helices. The first part of the beta sheet formed by S1, S2 and the N-terminal part of S3 is rather flat, while the second part involving S3, S4 and S5 is curved and twisted. In the loop between S1 and S2, there is a short 3^10^ helix H7. The long strand S3 contains a beta bulge, in which, between the amides of residues 149 and 153, three amino acids are outside the sheet, opposite to residue 167 in S4. This contributes to the sharp curvature of the sheet that is also stabilized by a zinc ion at the tip of the loop between S3 and S4. The last beta hairpin S4–S5 extends in the same direction as the lysine-rich loop, ordered by a crystal contact with the N-terminal domain of a symmetry-related molecule, but is disordered in three of the FusB copies. The C-terminus of FusB forms two helices H8–H9 in a 50° V-shape enclosed by the beta sheet. The domain contains a number of exposed conserved residues, mostly at the far end of the molecule.

**Figure 2.**
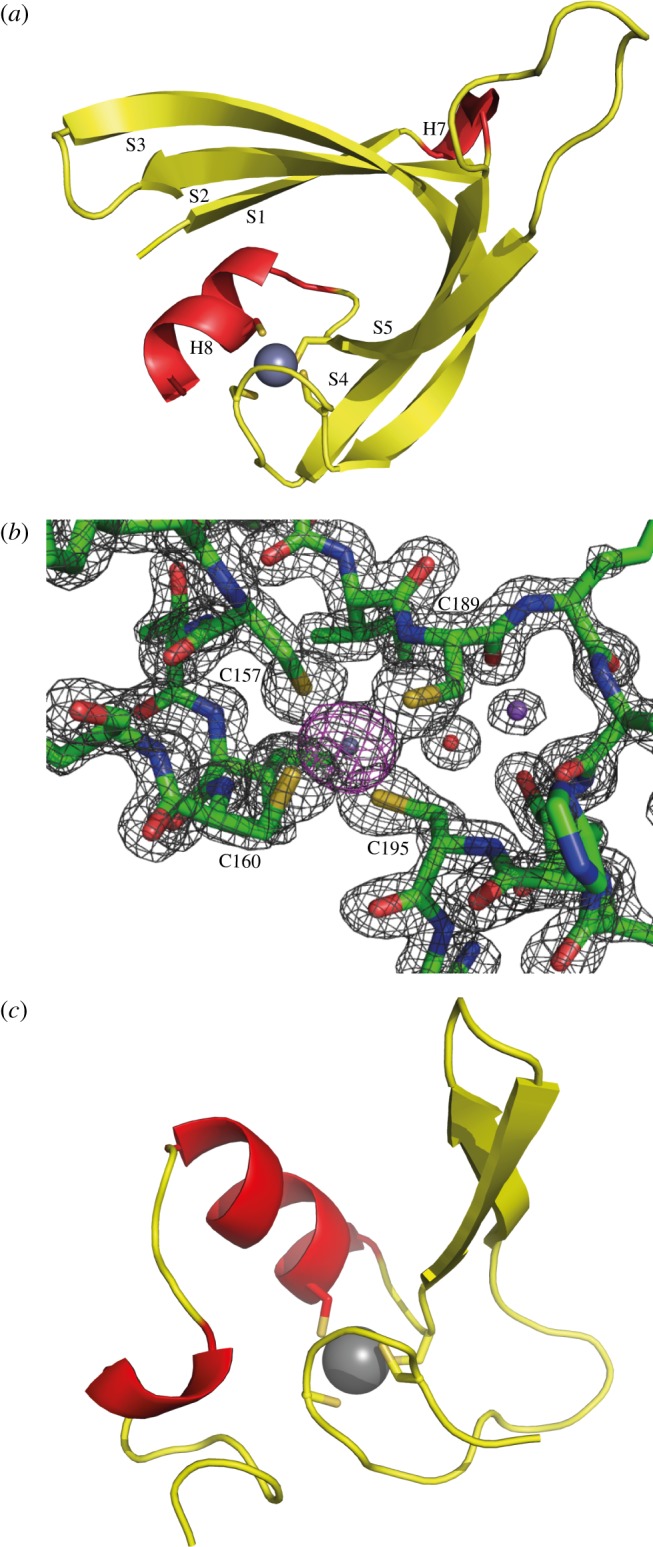
Structure of the FusB zinc site. (*a*) Structure of the FusB treble-clef zinc finger. Residues 127–208 are included, Zn is shown in grey and the four Cys ligands are shown as sticks. (*b*) Structure of the zinc site. The final 2F_o_-F_c_ map is contoured at 2.0 sigma (grey) and the anomalous difference map from a dataset at 1.278 Å wavelength is contoured at 4.0 sigma (magenta). Water is shown in red and sodium in purple. (*c*) Structure of ribosomal protein L24e including a standard treble-clef zinc finger (pdb 1vq8 [32]).

Beta strands S3–S5 form a non-canonical treble-clef zinc finger motif [31] (figure 2*a*,*b*), where, in this case, a zinc ion is coordinated by four strictly conserved cysteine residues in a CXXC-X23-CX5C motif (figure 1*c*). Out of the four zinc-coordinating residues, two are located in the loop between S3 and S4 (the so-called zinc knuckle), one at the end of S5 and one in H8. The zinc site forms a small and rigid core that stabilizes the overall structure of the C-terminal domain and the interface to the N-terminal domain. The orientation between the secondary structure elements is different when compared with the standard motif, exemplified with ribosomal protein L24e (figure 2*c*), as the polypeptide before the zinc knuckle forms a beta strand in the same sheet as the beta hairpin between the second and third cysteine. Additionally, the space between the two last cysteines is larger than the usual two to three amino acids.

### 3.5. Structural comparison

The two molecules in the *P*2_1_2_1_2 crystal form superpose with a root-mean-square deviation (RMSD) of 0.41 Å for 200 CA atoms while the two molecules in the *P*1 crystal form superpose with an RMSD of 0.49 for 197 CA atoms. When all four molecules were overlaid based on the C-terminal domain (residues 1–81), a minor rotational movement of the C-terminal domain could be visualized (electronic supplementary material, figure S2*a*). The maximum shift is 2.4 Å in the position of the H7 helix between molecule A in the *P*2_1_2_1_2 crystal form and molecule B in the *P*1 crystal form. One possible reason for the lack of conformational differences between the two crystal forms is the high similarity in crystal contacts involving the inside and outside of the domain border (electronic supplementary material, figure S3). The interface between the two domains is partly hydrophobic (electronic supplementary material, figure S2*b*) and trials to express the two domains separately failed, suggesting that the domains are not soluble as separate entities.

The region 172–185 has different conformations in the two molecules in the asymmetric unit. In the first molecule it is a perfect beta hairpin; in the other molecule, the two strands are separated from each other, so that Ala 188 has moved by 9.7 Å and residues 182–186 are disordered (electronic supplementary material, figure S2*a*).

### 3.6. Mapping of the FusB binding site on elongation factor G

**Figure 3.**
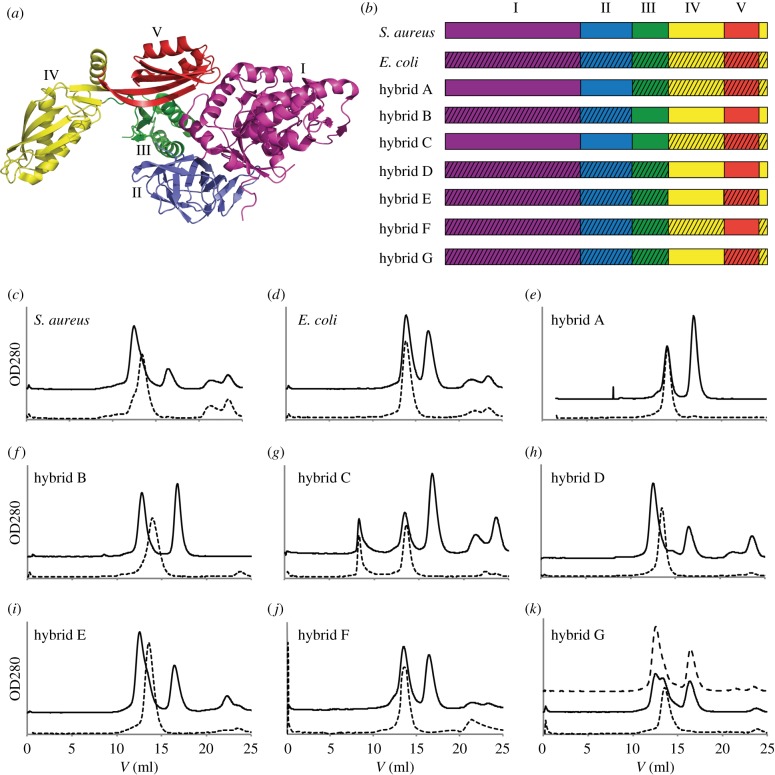
Mapping of determinants for FusB recognition of *S. aureus* EF-G. (*a*) Structure of *S. aureus* EF-G (pdb 2xex [10]) with domains indicated in different colours and by roman numerals. (*b*) EF-G constructs used in gel-filtration binding assays. Domains are coloured as in (*a*) and striped areas indicate *E. coli* sequence (remaining parts have *S. aureus* sequence). (*c*)–(*k*) Gel-filtration chromatograms of all EF-G constructs in (*b*) (dotted lines) and the same constructs in the presence of twofold excess of FusB (solid lines) on HiLoad 10/300 Superdex200. (*k*) Upper dotted line includes 2.5-fold excess of FusB.

FusB has previously been shown to form a complex with EF-G from *S. aureus*, but not with EF-G from *E. coli* [23]. Since these two EF-Gs share a sequence identity of 60 per cent and *S. aureus* EF-G is active in translation with *E. coli* ribosomes (see below), we decided to map the FusB binding site on EF-G using hybrid constructs, where some of the five domains were from *S. aureus* EF-G (figure 3*a*) and the rest from *E. coli* EF-G. Initially, four such constructs were made with either domains I and II from one species and domains III–V from the other species, or domains I–III from one species and domains IV and V from the other species (hybrids A–D, figure 3*b*). Wild-type and hybrid EF-Gs were tested for binding to FusB using size-exclusion chromatography. Because of the small difference in molecular weight of the EF-Gs and FusB–EF-G complexes (82–88 and 108–114 kDa, respectively), the experiments were performed with twofold excess of FusB so that the shift in elution volume of the EF-G peak could be used as a read-out of FusB binding. The shift in elution volume agrees with the increase in molecular mass upon formation of a 1 : 1 complex of EF-G and FusB. The large difference in height of EF-G peaks relative to FusB is explained by the different number of aromatic residues in the two EF-Gs leading to a 22 per cent higher theoretical extinction coefficient for *E. coli* EF-G.

Size-exclusion chromatography showed that FusB formed a complex with *S. aureus* EF-G as well as with hybrids B and D, where domains IV and V had *S. aureus* sequence (figure 3*c–h*). Further binding tests with hybrids E and F where only domain IV or domain V had *S. aureus* sequence (figure 3*b*) showed that domain IV contained the sequence determinants necessary and sufficient for FusB binding (figure 3*i*,*j*).

Finally, in hybrid G, we mutated the C-terminal helix of hybrid E (part of domain IV, but after domain V in the sequence; figure 3*a*,*b*) back to *E. coli* sequence. This construct displayed partial binding to FusB (figure 3*k*), indicating that this helix constituted part of the FusB binding site. To check that this construct was not partially misfolded, we also tested a higher FusB concentration and confirmed that EF-G could be saturated with FusB.

### 3.7. FusB-mediated rescue of fusidic acid inhibition in a reconstituted transcription–translation system

**Figure 4.**
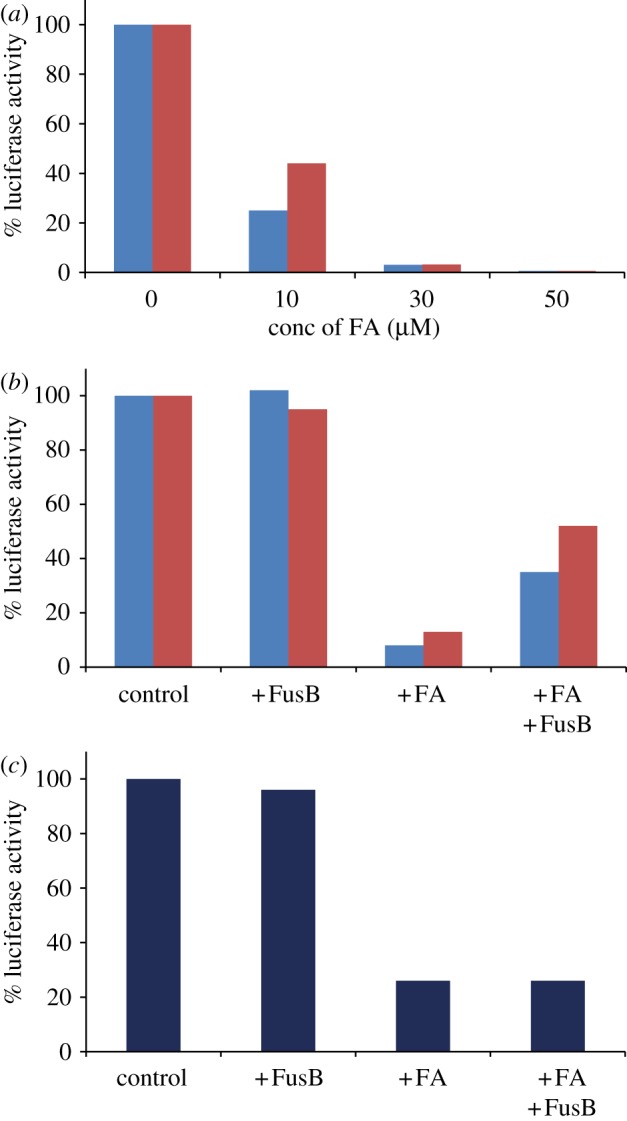
FusB action on FA-inhibited elongation and ribosome recycling. (*a*) The effect of FA (0–50 µM) on luciferase synthesis in a reconstituted transcription–translation system in the presence (blue bars) or absence (red bars) of RRF. EF-G was from *S. aureus* and all other components were from *E. coli.* (*b*) The effect of FusB (1 µM) on multiple round luciferase synthesis (with RRF, blue bars) or single round luciferase synthesis (without RRF, red bars) in the absence and presence of FA (20 µM). (*c*) The effect of FusB (1 µM) on multiple round luciferase synthesis using *E. coli* EF-G in the absence and presence of FA (20 µM).

We used a reconstituted transcription–translation system made up of purified translation components from *E. coli* [33] producing firefly luciferase to study the effect of FA and FusB in the EF-G-mediated steps of translation. Synthesis of luciferase was followed in a GloMax 20/20 luminometer for 1 h and the amount of active protein produced was estimated in luminescence units. The luciferase construct contained 593 amino acids and successful production thus required 592 translocation events. To allow only single-round luciferase synthesis, RRF was omitted from the reaction mixture. *Staphylococcus aureus* EF-G showed successful synthesis of luciferase with or without ribosome recycling (figure 4*a*, no FA), with approximately half the yield compared with *E. coli* EF-G (data not shown), demonstrating its compatibility with an *E. coli*-based translation system. The luciferase yield gradually decreased with increasing concentration of FA (figure 4*a*). Interestingly, the reaction was inhibited more efficiently including ribosome recycling than without (75% and 60% inhibition, respectively) at an FA concentration of 10 µM.

We tested the effect of FusB on FA inhibition in multiple- and single-round luciferase synthesis with *S. aureus* EF-G (figure 4*b*). In the former reaction, EF-G drove both elongation and recycling, whereas in the latter case ribosome recycling was excluded. Addition of FusB increased both multiple- and single-round luciferase synthesis (figure 4*b*) up to four- to fivefold when compared with the reactions with only FA. Thus, these experiments demonstrated that FusB could provide FA resistance in elongation as well as in ribosome recycling. FusB could not rescue FA inhibition of luciferase synthesis with an identical system including *E. coli* EF-G (figure 4*c*), in line with a previous report [23]. However, as shown in figure 4*b*,*c*, the activity of FusB was not dependent on the origin of any other translational component except *S. aureus* EF-G.

### 3.8. FusB-mediated rescue of fusidic acid inhibition of a single elongation step

**Figure 5.**
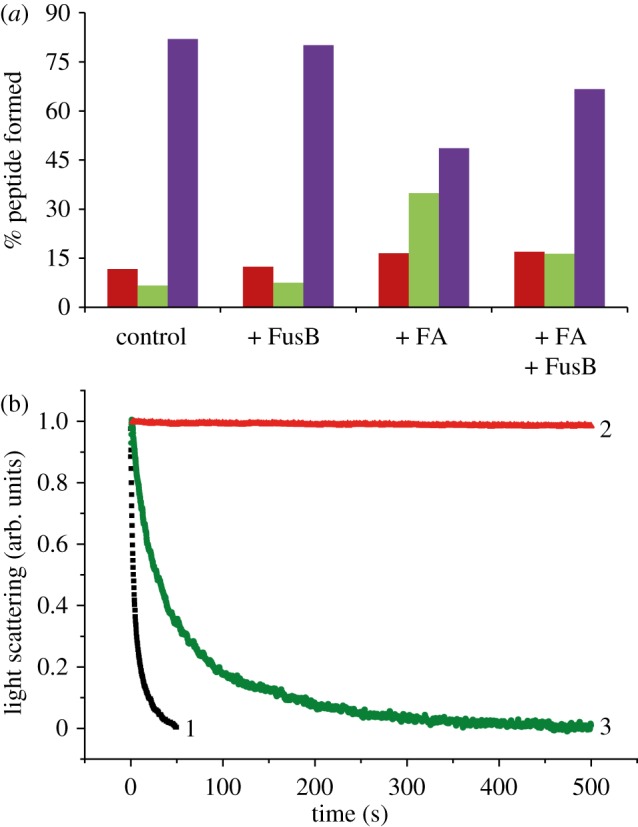
The effect of FA and FusB on tripeptide formation and ribosome recycling. (*a*) Tripeptide formation was initiated by mixing IM and EM containing *S. aureus* EF-G (see §5.10 for details). The fraction of mono- (fMet, red bars), di- (Met-Phe, green bars) and tri- (Met-Phe-Phe, purple bars) peptide formed in 10 s without (control) or with different combinations of FA (150 µM) and FusB are presented as indicated below the bars. (*b*) Splitting of a post-termination complex (0.5 µM) (programmed with MFF-stop mRNA) was followed by decrease in Rayleigh light scattering by mixing with RRF (10 µM), and *S. aureus* EF-G (5 µM) in the stopped flow without FA and FusB (trace 1), with FA (10 µM) alone (trace 2) or with FA (10 µM) and FusB (1 µM) (trace 3).

We have tested the effect of FusB on a tripeptide formation assay using *S. aureus* EF-G. The reaction was quenched in 10 s, which was long enough for the completion of a single elongation step, but too short for multiple rounds. FA (150 μM) inhibited tripeptide formation to 50 per cent, and in the presence of 1 μM FusB the extent of tripeptide formation recovered to approximately 75 per cent (figure 5*a*). FusB alone at the same concentration did not influence the yield. The observed rescue was specific to *S. aureus* EF-G, as no rescue occurred with *E. coli* EF-G (data not shown). These results confirmed that FusB rescued EF-G function from FA inhibition in the peptide elongation step.

### 3.9. FusB-mediated rescue of fusidic acid inhibition of ribosome recycling

Splitting of a post-termination complex by EF-G and RRF was monitored in a stopped-flow instrument with Rayleigh light scattering, where the smaller size of the ribosomal subunits compared with 70S leads to a decrease in light scattering. *Staphylococcus aureus* EF-G, together with *E. coli* RRF, could successfully split *E. coli* 70S at a rate of 0.15 s^−1^ (figure 5*b*). The splitting reaction was completely blocked with 10 μM FA and no reaction was observed even at longer incubation. When FusB (1 μM) was added to the FA-inhibited reaction, ribosome splitting could occur again, although at a lower rate (0.02 s^−1^), demonstrating that FusB could rescue FA-mediated inhibition of ribosome splitting driven by EF-G and RRF.

## 4. Discussion

### 4.1. FusB structure

We have solved the crystal structure of FusB in two different crystal forms at different pH. The structure has two domains, where the N-terminal four-helix bundle is a common fold that occurs in proteins with many different functions. It provides a rigid structure stabilized by a tightly packed hydrophobic core in combination with salt bridges and hydrogen bonds between neighbouring helices.

The C-terminal domain has a unique fold stabilized by a treble-clef-like zinc finger motif. These motifs display limited sequence similarity and occur in proteins involved in various functions, but often interacting with nucleic acids [31]. Apart from the zinc site, FusB does not display significant similarity to any other protein of this class.

After submission of the present work, we became aware of a very recent paper describing the crystal structure of the FusB homologue FusC (42% sequence identity to FusB; figure 1*c*) and the biochemical characterization of how FusB acts on non-programmed ribosome complexes with and without FA [34]. The crystal structure of FusC (pdb 2yb5) is very similar to the structures of FusB. The entire structure superimposes with RMSD of 1.4 Å (for 194 CA atoms) or less onto any of the four FusB structures. When the FusB and FusC structures are superimposed based on domain I, there is a 2.2 Å shift of the Zn ion (electronic supplementary material, figure S2*c*).

FusB binds with high affinity to EF-G and also has propensity to bind nucleic acids, as observed during purification. Analysis of the sequence conservation mapped on the structure (figure 1*c*–*e*) suggests that both of these binding activities are likely to reside in the C-terminal domain. The exposed conserved residues in the C-terminal domain cluster in three regions—the H5–H6, S1–S2 and S4–S5 loops—all located at the far end of FusB (figure 1*c*–*e*) and potentially contributing to the same macromolecular binding site. Secondary structure prediction suggested the H5 helix would continue until residue 102, including most of the Lys residues that now project away from each other. A longer helix may form upon interaction with a negatively charged binding partner, and this may lead to a shift in the position of H5 with respect to S2 and S3 or induce an inter-domain movement. We observe different conformations of the S4–S5 loop, but no major inter-domain movements between the two crystal forms; however, we cannot exclude that larger changes would occur upon binding to, for example, EF-G.

The function of nucleic acid binding by FusB is unknown, but two possibilities are an interaction with the ribosome or involvement in the regulation of its own synthesis. FusB is induced by translational attenuation based on an alternative mRNA structure that is formed only when ribosomes are stalled on the *fusB* leader sequence during FA inhibition [23].

Additional candidate EF-G-binding regions are the two sides of the inter-domain interface that demonstrate their protein-binding propensity in crystal packing, and where we observe an unknown density (electronic supplementary material, figure S1). To create specific protein–protein binding, a binding surface around 1000 Å^2^ is normally needed [35], and the crystal packing interactions involve approximately this area. In the recent paper by Cox *et al.*, the EF-G binding site on FusB was mapped through nuclear magnetic resonance (NMR) chemical shift mapping of backbone amides, showing that EF-G probably binds to the beta strands S3, S4 and S5 [34] (upper part of beta sheet in figure 1*a*, top). Part of this surface is involved in forming the ‘hugging’ dimer that we observe in crystal packing (electronic supplementary material, figure S3). However, since the loop regions H5–H6, S1–S2 and S4–S5 at the far end of the molecule were not assigned in the NMR experiment, it does not provide any data regarding their potential involvement in EF-G binding [34]. Interestingly, there were also chemical shift differences in the inter-domain linker that could be a sign of inter-domain movement.

### 4.2. *Staphylococcus aureus* elongation factor G works with *Escherichia coli* translation components

Gram-positive *S. aureus* is distantly related to gram-negative *E. coli*. Our biochemical experiments show that *S. aureus* EF-G can still function with *E. coli* ribosomes *in vitro*. The 60 per cent sequence identity between EF-G from these two species must preserve functionally critical interactions between EF-G and the ribosome. Also, even though FusB binding and rescue are specific to *S. aureus* EF-G, FusB can exhibit its protective activity when all other components are from *E. coli* (figure 4*c*).

### 4.3. FusB acts in elongation and recycling

GTP hydrolysis by EF-G is used to drive elongation as well as ribosome recycling together with RRF. FA acts by inhibiting the release of EF-G·GDP from the ribosome in both of these steps. It was shown that FA inhibits ribosome recycling more effectively than elongation [14]. Similarly, in our experiments (figure 5), FA inhibition of recycling occurs at a lower drug concentration than elongation, although the difference in the FA concentration was smaller than reported earlier. Also, in the reconstituted transcription–translation system with more than 500 rounds of elongation and one round of ribosome splitting per luciferase molecule, there is a notable difference in inhibition with and without RRF (figure 4*b*,*c*).

It is an open question whether FusB would rescue either or both of the steps involving EF-G. The only previous information available regarding FusB activity was from experiments conducted in a staphylococcal S30 extract [23]. Our experiments with *S. aureus* EF-G in a reconstituted transcription–translation system (*E. coli*) enabled us to test its function with or without ribosome recycling. FusB recovered luciferase synthesis from FA inhibition in the presence as well as in the absence of RRF (figure 4*b*). These results were further confirmed by fast kinetic measurements of elongation and ribosome splitting under single turnover conditions, where FusB rescued both reactions fully or partially from FA inhibition (figure 5*a*,*b*). Thus, we demonstrate for the first time that FusB rescues the translation system from FA inhibition in both the elongation and recycling steps, representing EF-G locked to ribosomes in classical and ratcheted states, respectively [14].

### 4.4. FusB binding site on elongation factor G: implications for function

We have located the determinants for FusB binding of *S. aureus* EF-G to domain IV and showed that the terminal helix of EF-G forms a part of this binding site. However, we cannot exclude that FusB might make additional interactions with other parts of EF-G that are conserved between *S. aureus* and *E. coli*. In any case, as judged by size-exclusion chromatography, a hybrid construct containing domain IV of *S. aureus* EF-G and the remaining sequence from *E. coli* shows the same degree of complex formation with FusB as the wt *S. aureus* EF-G. We estimate that *K*_d_ for the FusB–EF-G complex is in the low micromolar range, or lower, and we fail to detect any difference in affinity between FusB and EF-G in the presence of GDP, GTP or FA (data not shown). To gain further information regarding the interactions, we would need to check the affinity using other methods such as Biacore or ITC. Cox *et al.* [34] have determined the affinity between FusB and EF-G, as well as FusB and domains III–V of EF-G, to 60 nM using ITC.

Domain IV of EF-G is composed of two segments: residue 481–603 and the C-terminal helix 675–693 (*S. aureus* numbering). The terminal helix makes contact with domain IV as well as domain V, and accommodates to maintain both interactions when EF-G changes its conformation, as judged by comparing crystal structures of *T. thermophilus* EF-G in isolation [12] and on the ribosome locked with FA [6]. The difference in FusB binding by EF-G hybrids E and G (figure 3) could be owing to surface properties of the helix or interactions of the terminal helix with domains IV and V that disturb binding of FusB, but shows that the helix directly or indirectly is important for the interaction of FusB with *S. aureus* EF-G.

### 4.5. Relevant mechanisms of antibiotic resistance

There are four common mechanisms of antibiotic resistance. The first one is active efflux involving, for example, multi-drug resistance transporters or decreased drug uptake. The second one is modification of the drug target (e.g. the point mutations of residues in EF-G that directly interact with FA that cause high-level resistance [6]). Third, antibiotics can be enzymatically degraded or modified. Fourth, an organism can evolve an altered metabolic pathway to circumvent the drug-caused inhibition. To our knowledge, there are very few examples in the literature of resistance mechanisms that do not fall into these four categories.

Tetracyclin is a translation-inhibiting antibiotic for which one of the resistance mechanism involves so-called ribosomal protection proteins (RPPs; reviewed in [36]). Tetracycline binds to 16 S rRNA close to the decoding centre, preventing binding of aminoacyl-tRNA to the A site. The RPPs such as Tet(M) and Tet(O) are sequence homologues of EF-G that release tetracycline from the ribosome in a GTP-dependent manner [37], probably by locally disturbing the rRNA structure of the binding site, as indicated by cryo-electron microscopy [38].

Quinolones and fluroquinolones bind to DNA complexes of DNA gyrase and topoisomerase IV, and trap enzyme complexes with cleaved DNA. Pentapeptide-repeat protein (PRP) MfpA in mycobacteria and the analogous Qnr proteins in enterobacteria cause a low level of quinolone resistance. These resistance proteins form DNA-mimicking structures [39], which were suggested to destabilize the drug-inhibited complexes and to cause release of the antibiotic and the DNA [40].

Thus, a fifth general resistance mechanism unifying the tetracycline RPPs and the quinolone PRPs might be to increase the off-rate of the antibiotic by binding to and altering the conformation of the target. If FusB were to act by a similar mechanism, it would imply that FusB would increase the off-rate of FA and/or of EF-G in the presence of FA. The latter was demonstrated to occur on vacant ribosomes (lacking mRNA and tRNAs), where FusB accelerated the dissociation of EF-G in the presence as well as in the absence of FA [34], suggesting that FusB acts according to the same mechanism on programmed ribosomes *in vivo*.

### 4.6. Suggested mechanisms of FusB action

FA binds to a pocket between domains I, II and III of EF-G in the post-translocational state with GDP on the ribosome. By stimulating the release of EF-G [34], FusB can release this inhibition so that the ribosome can accept a new aminoacylated tRNA or split into subunits, allowing translation to proceed.

In our experiments, we demonstrate FusB-mediated rescue at 1 µM concentration, but the concentration of FusB in *S. aureus* under induced conditions remains unknown. The *K*_d_ of the FusB–EF-G complex is around 60 nM [34], and EF-G and ribosomes are present at roughly 1 µM concentration *in vivo*, suggesting that all free EF-G may be bound to FusB. Still, two scenarios are possible: FusB may cycle between EF-G molecules or stay bound to EF-G during translation.

While *fusA* mutants typically have poor growth rate when compared with wild-type and acquire secondary fitness-compensatory mutations [17,41], expression of FusB or its homologues FusC and FusD does not affect the growth rate in the absence of FA [25], suggesting that these proteins do not have a negative effect on EF-G activity. Thus, if FusB stays bound to EF-G, the complex should have a similar level of activity as the free EF-G.

Would an interaction of FusB with domain IV of EF-G be compatible with ribosome binding? Apart from the terminal helix, we do not know which parts of domain IV, and possibly of other domains, are involved in FusB binding. On the ribosome, only the tip of EF-G domain IV makes contact with mRNA, P-site tRNA and the decoding centre [6], and the surface with the beta sheet (figure 4*a*) is accessible from the outside, and potentially available for binding to FusB. The terminal helix does not make any direct contact with the ribosome in the FA-locked state. However, to reach this helix when EF-G is bound to the ribosome, FusB would need to insert between domain IV of EF-G and the P-site tRNA, a space surrounded by 23 S rRNA helix 38, L11 and L25. A recent docking experiment [34] suggests that FusB binds to a surface of domains III and IV of EF-G that is inaccessible on the ribosome, which we consider unlikely, as FusB can accelerate the release of EF-G [34].

From the present data, we thus propose two possible mechanisms: (1) FusB binds to EF-G in the FA-locked state and induces conformational changes that facilitate dissociation of EF-G and FA; (2) the FusB–EF-G complex performs translocation and recycling on the ribosome, but FusB prevents EF-G from reaching the conformation to which FA binds or prevents locking in the presence of FA.

The conformational changes needed for FA dissociation could be on the inter-domain level or local changes in the FA-binding site. There are several examples of *fusA* resistance mutations that are predicted to cause resistance by affecting the inter-domain arrangement in EF-G [10]. If FusB would bind to EF-G using the beta sheet S3–S4–S5, as indicated by chemical shift mapping [34], or the conserved loops at the outer edge of the C-terminal domain, the rigid N-terminal domain could provide a lever interacting with another part of EF-G or with the ribosome to achieve conformational change of EF-G. The distance between the terminal helix and FA is roughly 40 Å (pdb 2wri [6]), which would allow FusB to bridge between the two relevant sites.

To further clarify the mechanism of FusB-mediated FA resistance, we will pursue our biochemical studies, as well as structural studies of the FusB–EF-G complex. Here, we have solved the crystal structure of FusB, mapped the sequence determinants for its recognition of EF-G, and monitored FusB-dependent rescue of FA inhibition in translocation and recycling, providing a solid foundation for further experiments.

## 5. Materials and methods

### 5.1. Sequence analysis

Sequence alignment was done using the CLUSTALW2 server [42].

### 5.2. Cloning of FusB

A single colony of *S. aureus* WBG157, pUB101 (gift from D. Hughes), was grown in 15 ml Luria broth (LB) medium containing 0.5 per cent glycine at 37°C overnight without shaking. The pelleted cells were resuspended in 100 µl lysis buffer (20 mM Tris pH 7.5, 0.1 g ml^−1^ lysostaphin, 30 mg ml^−1^ lysozyme, 3 mg ml^−1^ proteinase K) and incubated at 37°C for 1 h. Starting from the lysate, pUB101 was purified using the QIAprep Spin Miniprep kit (Qiagen). To obtain a FusB construct with an N-terminal His-tag, the *fusB* gene was amplified by PCR using PfuUltra high-fidelity DNA polymerase (forward and reverse primers: 5′-ATGGCTCATCATCATCATCATCATGGTATGAAGACAATGATTTATCCTCAC-3′ and 5′-CACAAACATAGTTAATTCCTTAATCTAG-3′, respectively) using pUB101 as a template. The PCR product was cloned into pEXP5-CT/TOPO (Invitrogen). The correctness of the pFusB-SN construct was confirmed by sequencing.

### 5.3. FusB expression and purification

pFusB-SN was transformed into BL21(DE3) cells and plated on a Luria agar (LA) plate containing 50 μg ml^−1^ ampicillin. A single colony was added to LB medium with 50 μg ml^−1^ ampicillin and grown at 37°C o/n. One litre of culture was inoculated with 5 ml o/n culture and incubated at 100 r.p.m. at 37°C. One millimolar isopropyl β-D-1-thiogalactopyranoside (IPTG) was added at OD (600) of 0.5. After 5 h, the cells were pelleted by centrifugation at 4000*g* for 30 min. The cell pellet was resuspended in lysis buffer (50 mM Tris pH 7.5, 300 mM NaCl, 20 mM imidazole) supplemented with Complete Mini EDTA-free protease inhibitor (Roche). The cells were lysed by sonication and the debris was pelleted at 18 000 r.p.m. for 30 min. The supernatant was transferred into an Econo-Pac gravity column (Bio-Rad) together with 2 ml Ni Sepharose (GE Healthcare) and equilibrated at 4°C for 30 min. The matrix was washed with wash buffer (50 mM Tris pH 7.8, 600 mM NaCl, 20 mM imidazole) and FusB was eluted in elution buffer (50 mM Tris pH 7.8, 300 mM NaCl, 400 mM imidazole) and further purified on a Hiload 16/60 Superdex75 column (GE Healthcare) equilibrated in gel filtration buffer (20 mM Tris pH 7.8, 300 mM NaCl). The FusB peak was concentrated to 16 mg ml^−1^ in a VIVASPIN 6 (Sartorius Stedim) with a membrane cut-off of 10 000 D.

### 5.4. Crystallization

Initial screening of crystallization conditions was performed at the High Throughput Crystallisation Laboratory, Grenoble, France. Hits from the Index screen (Hampton Research) were optimized by varying the pH and precipitant concentration. Diffraction quality crystals were obtained using the hanging-drop vapour diffusion method at 4°C. The drops contained 2 μl protein solution and 2 μl reservoir solution (200 mM (NH_4_)_2_SO_4_, 100 mM Bis–Tris pH 5.5, 18% (w/v) PEG3350). Crystals grew to a size of 0.05 × 0.05 × 0.3 mm in two weeks and were cryo-protected in reservoir solution supplemented with 20 per cent (v/v) glycerol, and vitrified in liquid nitrogen. For the iodine derivative, crystals were soaked in cryo-protectant solution including 1 M of NaI for 4 h.

A second crystal form was obtained by the same method using a reservoir solution containing 200 mM (NH_4_)_2_SO_4_, 100 mM Mops-Tris pH 8.1 and 25% (w/v) PEG3350. At this condition, crystals grew to a size of 0.03 × 0.03 × 0.005 mm in one month.

### 5.5. Structure determination

Native and derivative datasets of FusB crystals were collected at beamlines ID23-1 and ID23-2 (ESRF, Grenoble) at 100 K. The iodine SAD dataset was collected in six 60° wedges, each collected from a new section of the elongated crystal. Data were processed with the XDS package [43]. The crystals belong to space group *P*2_1_2_1_2; X-ray data statistics are summarized in table 1. Nineteen iodine sites could be located in SAD phasing using Phenix [44] with a figure-of-merit of 0.449. Automatic building of the structure containing two FusB molecules in the asymmetric unit followed by rebuilding against the high-resolution native data was performed in Phenix. Further cycles of manual building were performed in Coot [45] and refined using Phenix. The final structure containing the complete FusB sequence (except the His-tags and residues 172–183 in the B molecule) was refined to *R*_work_ 16.3 per cent and *R*_free_ 20.2 per cent.

**Table 1.** Functional classification of differentially expressed genes in *Mtb* HN878-infected rabbit lungs (percentage).^a^

	FusB1	FusB iodine	FusB2
data collection statistics			
beamline	ID14-4	ID23-1	ID23-1
wavelength (Å)	0.9392	1.5498	0.9537
space group	*P*2_1_2_1_2	*P*2_1_2_1_2	*P*1
cell dimensions			
*a*, *b*, *c* (Å)	74.98, 122.28, 52.89	74.85, 120.60, 52.76	45.10, 47.61, 53.24
*α*, *β*, *γ* (°)	90, 90, 90	90, 90, 90	89.76, 83.46, 85.76
resolution (Å)^a^	48.58–1.65 (1.70–1.65)	50–2.5 (2.60–2.50)	50–2.30 (2.35–2.30)
*R*_merge_^b^	8.7 (53.9)	6.9 (11.3)	7.3 (43.6)
〈 *I*/*σ*(I) 〉	22.2 (4.0)	24.7 (15.4)	14.4
completeness (%)	98.1 (86.1)	97.8 (92.0)	94.5 (95.9)
redundancy	14.9	7.5	3.9
refinement statistics			
reflections (test set)	58 343 (2918)		23 932 (905)
number of protein atoms	3648		3439
number of waters	353		99
B-factor (Å^2^)			
protein	27.7		48.3
waters	35.4		36.8
*R*_work_/*R*_free_ (%)	16.3/20.2		20.4/25.4
RMSD from ideal bond length (Å)	0.011		0.018
RMSD from ideal bond angle (°)	0.873		1.605
Ramachandran plot			
preferred (%)	98.9		97.3
allowed (%)	1.1		2.7
outliers (%)	0		0

^a^Values in parentheses represent the highest resolution bin.

^b^
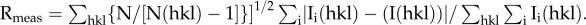

Data from the second crystal form were processed with the XDS package [43] in space group *P*1. The structure was solved by molecular replacement in Phaser [46] using the *P*2_1_2_1_2 FusB structure as search model. The asymmetric unit contained two FusB molecules in similar arrangement as in the *P*2_1_2_1_2 crystals. The structure containing the complete FusB sequence except the His-tags, residues 176–179 in the A molecule, and residues 103–104, 174–181 and 213 in the B molecule, were refined to *R*_work_ 20.4 per cent and *R*_free_ 25.4 per cent using Phenix [44]. The quality of the structures was assessed in Phenix. The refinement statistics can be found in table 1. The refined coordinates have been deposited in the protein data bank with accession numbers 4adn (*P*2_1_2_1_2) and 4ado (*P*1). All structural figures were prepared using PyMOL [47].

### 5.6. Cloning and preparation of hybrid elongation factor G constructs

All hybrid EF-G constructs were made by cloning the respective *E. coli* sequence into the vector pET30-Sa-EFG [48] by restriction-free cloning [49]. First, the DNA sequence encoding the desired *E. coli* domain(s) was amplified using a plasmid construct of *E. coli* EF-G as template. The primers were designed so that the resulting DNA fragment had the *E. coli* sequence of choice flanked with extensions complementary to the sequence around the insertion site in the vector with the *S. aureus* EF-G encoding sequence (electronic supplementary material, table S1). In a second, linear amplification reaction, this DNA fragment was used as primer pair, and the pET30-Sa-EFG vector was used as template. Construct pEFG-ECO1-615_SAU604-674_ECO687 was done with two PCR products introduced simultaneously into the vector [50]. All amplifications were done using PfuUltra (Stratagene). The resulting DNA was treated with *Dpn*I to digest the parental vector and transformed into OneShot TOP10 Chemically Competent *E. coli* (Invitrogen). The correctness of the hybrid EF-G constructs was verified by DNA sequencing.

For expression, all hybrid EF-G plasmid constructs were transformed into BL21(DE3) cells and plated on LA plates containing 50 μg ml^−1^ kanamycin at 16°C o/n. One litre cultures in LB medium were inoculated with 5 ml o/n culture and grown at 37°C until OD(600) of 0.5. The cultures were cooled before addition of 1 mM IPTG and further incubation at 16°C o/n. Purification was done using the same protocol as for FusB but using EF-G wash buffer (50 mM Tris–HCl, 200 mM NaCl, 20 mM imidazole, 5 mM 2-mercaptoethanol, pH 7.5) and EF-G elution buffer (50 mM Tris–HCl, 200 mM NaCl, 500 mM imidazole, 5 mM 2-mercaptoethanol, pH7.5). Size-exclusion chromatography was performed using a HiLoad 16/60 Superdex200 column (GE Healthcare).

### 5.7. Size-exclusion binding assay

Samples of 2 nmol EF-G (wild-type or hybrid constructs) with or without 4 nmol FusB were prepared in 100 μl gel filtration buffer and loaded on a Hiload 10/300 Superdex200 column (GE Healthcare) equilibrated in the same buffer. Protein concentration was determined based on A280 and theoretical extinction coefficients (47 790 M^−1^ cm^−1^ for *S. aureus* EF-G and 58 460 M^−1^ cm^−1^ for *E. coli* EF-G; varying between these two values for the hybrid constructs). All constructs including domain III from *S. aureus* were aggregation-prone and had to be used fresh from gel filtration.

### 5.8. Components for biochemical experiments

All translation components except EF-G were from *E. coli.* EF-G from *S. aureus* was overexpressed and purified as described [10]. The MRE600 ribosomes, His-tagged *E. coli* translation factors, XR7 fMet-Phe-Phe-stop (MFF) mRNA and fMet-tRNA^fMet^ were purified as described earlier [33,51]. The experiments were performed in HEPES polymix buffer (pH 7.5) at 37°C (for details see [33]). Additionally, the reactions contained energy components such as ATP (1 mM), GTP (1 mM), phosphoenolpyruvate (PEP, 10 mM), pyruvate kinase (PK, 50 µg ml^−1^) and myokinase (MK, 2 µg ml^−1^). FA was from Leo Pharma (Denmark). tRNA^Phe^ was purchased from Chemical Block (Moscow, Russia). PEP, PK, MK and non-radioactive amino acids were from Sigma-Aldrich. Radioactive amino acids and nucleotide triphosphates were from GE Healthcare.

### 5.9. FusB action in a reconstituted transcription–translation system

*Staphylococcus aureus* or *E. coli* EF-G were used in a reconstituted transcription–translation system composed of *E. coli* components for synthesis of a 593 amino acid construct of firefly luciferase [33]. This system was used to study the effect of FA (10–50 µM) and FusB (1 µM) in various combinations. To study single-round synthesis of luciferase involving only the elongation function of EF-G, RRF was excluded from the reaction mix.

### 5.10. Tripeptide formation assay

The initiation complexes (IM) were formed by incubating 70S ribosomes (1 µM), [^3^H]fMet-tRNA^fMet^ (1 µM), XR7 mRNA fMet-Phe-Phe-stop (MFF) (4 µM), initiation factors: IF1, IF2, IF3 (1 µM each) at 37°C for 15 min. An elongation mix (EM) containing EF-Tu (10 µM), EF-Ts (5 µM), phenyl alanine (200 µM), tRNA^Phe^ (5 µM), tRNA^Phe^-synthetase (0.2 units µl) and EF-G (5 µM), without or with FA (150 µM) and FusB (1 µM) was also incubated at 37°C for 15 min. Equal volumes of IM and EM were mixed by hand and the reaction was quenched after 10 s by adding formic acid (17% final concentration). The ribosome-containing pellet was dissolved in potassium hydroxide, and the amount of MF di- and MFF tripeptide was analysed with reversed-phase high-performance liquid chromatography by comparing the respective peaks with the peak of [^3^H]fMet. All experiments were performed in triplicates.

### 5.11. Ribosome recycling assay

A post-termination complex was formed by incubating 70S ribosomes (0.5 µM), XR7 MFF mRNA (2 µM) and tRNA^Phe^ (3 µM) at 37°C for 20 min. To this complex, a mix containing RRF (10 µM), EF-G (5 µM) and IF3 (2 µM) without or with FA (10 µM) and FusB (1 µM) preincubated at 37°C was rapidly added in a stopped flow apparatus. The kinetics of splitting of 70S ribosomes into subunits was monitored by measuring Rayleigh light scattering as described [52].

## Supplementary Material

Supporting Online Material
