# Supplementary material for: Structure and function of FusB: an elongation factor G-binding fusidic acid resistance protein active in ribosomal translocation and recycling
Source: Open Biol. 2012 Mar;2(3):120016. doi: 10.1098/rsob.120016 (PMC3352095; doi:10.1098/rsob.120016)
Supplement: Supporting Online Material [file rsob120016-s1.pdf]

Supporting Online Material for

Structure and function of FusB – an EF-G binding fusidic acid resistance protein active in ribosomal translocation and recycling

Xiaohu Guo, Kristin Peisker, Kristina Bäckbro, Yang Chen, Ravi Kiran Koripella, Chandra Sekhar Mandava, Suparna Sanyal* & Maria Selmer*

* To whom correspondance should be addressed:

suparna.sanyal@icm.uu.se

maria.selmer@icm.uu.se

This pdf includes:

Table S1

Figures S1, S2 and S3

Supplementary references

Table S1 . Primers used in creation of hybrid EF-G constructs.

construct	*S. aureus* domains	*E.coli* domains	forward primer*	reverse primer*	template in linear amplification reaction
Hybrid A: pEFG-SAU1-397_ECO406	I, II	III, IV, V	*gtggtgagaaaaatgacattatc*CTGGAACGTATGGAATTCCCTGAG	*catggttgaggagaagcccggttta*TTTACCACGGGCTTCAATTACGGC	pET30-Sa-EFG
Hybrid B: pEFG-ECO1-405_SAU398	III, IV, V	I, II	*ctgggtaccgatgacgacgacaag*ATGGCTCGTACAACACCCATCG	*gctctgggaattccattgattccaa*AATGATCGGCGCATCCGGGTC	pET30-Sa-EFG
Hybrid C: pEFG-SAU1-480_ECO489	I, II, III	IV, V	*gtaacgtaggtgctccaatggtt*GCTTACCGTGAAACTATCCGCCAG	*catggttgaggagaagcccggttta*TTTACCACGGGCTTCAATTACGGC	pET30-Sa-EFG
Hybrid D: pEFG-ECO1-488_SAU481	IV, V	I, II, III	*ctgggtaccgatgacgacgacaag*ATGGCTCGTACAACACCCATCG	*gatttgaatgtttcacgatatga*AACCTGCGGTTTACCTACGTTCG	pET30-Sa-EFG
Hybrid E: pEFG-ECO1-488_SAU481-603_ECO616-686_SAU675-693	IV	I, II, III, V	*gtgatcctgtaatcttagaacca*ATCATGAAGGTTGAAGTAGAAACTCCG	*cgattgattttggaacttcagcata*CTTCAGGAATTCCATAGTGTATGATGC	EFG-ECO1-488_SAU481
Hybrid F: pEFG-ECO1-615_SAU604-674_ECO687-704	V	I, II, III, IV	PCR fragment a. *ctgggtaccgatgacgacgacaag*ATGGCTCGTACAACACCCATCG<br>PCR fragment b. *cggtacttacactatgtacttcgatcac*TATGATGAAGCGCCGAGTAACGTTG	PCR fragment a. *caggcatttcaatagttacttcatcat*CGGCTCAAGCAGAACTGGTTTC<br>PCR fragment b. *catggttgaggagaagcccggttta*TTTACCACGGGCTTCAATTACGGC	pET30-Sa-EFG
Hybrid G: pEFG-d4SAU_ECO687-704	IV - 675-693	I, II, III, V + 687-704	*gtgatcctgtaatcttagaacca*ATCATGAAGGTTGAAGTAGAAACTCCG	*catggttgaggagaagcccggttta*TTTACCACGGGCTTCAATTACGGC	pEFG-ECO1-488_SAU481-603_ECO616-686_SAU675-693

* The sequence complementary to the *E. coli* domain(s) is in capitals, the sequence complementary to the vector used in the linear amplification is in italics.

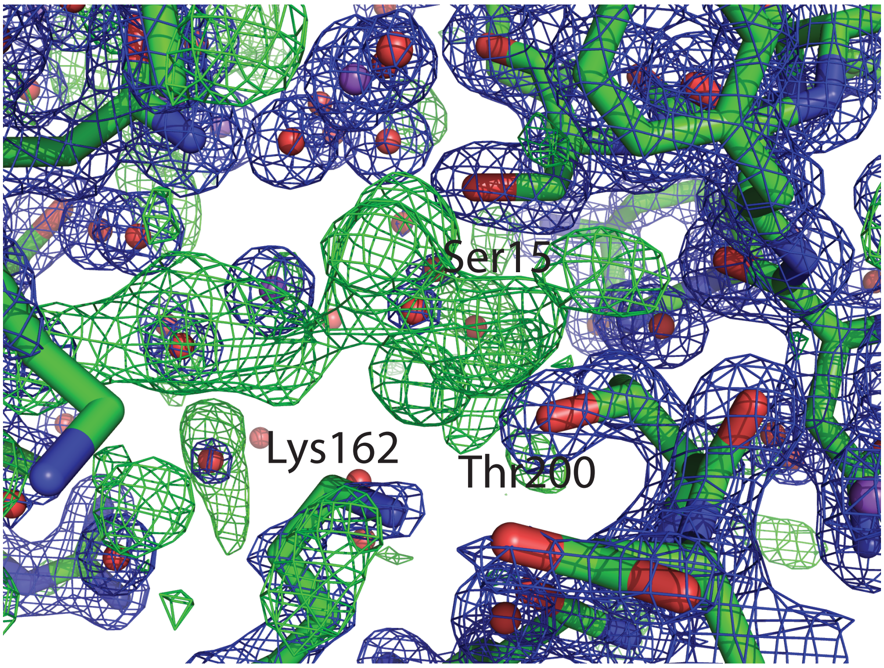

Figure S1. Un-interpreted electron density at the domain interface.

The refined $P2_12_12$ FusB structure (molecule A) is shown as green sticks. The final $2F_o$-F_c map is contoured at 2.0 sigma (blue) and the F_o-F_c map is contoured at 3.0 sigma (green).

(a)

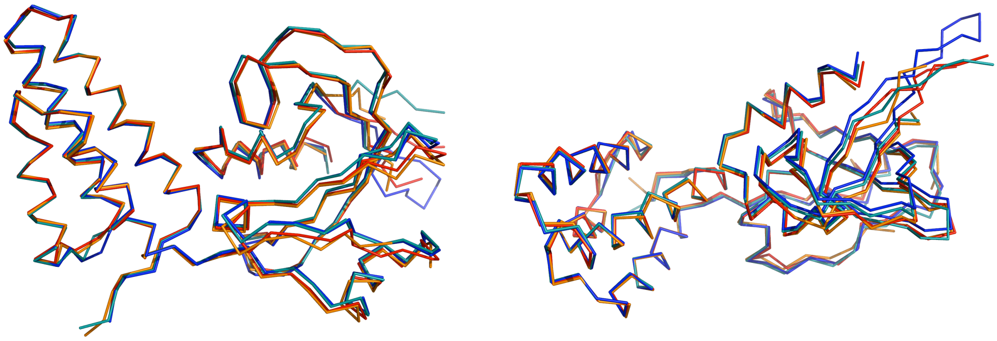

(b)

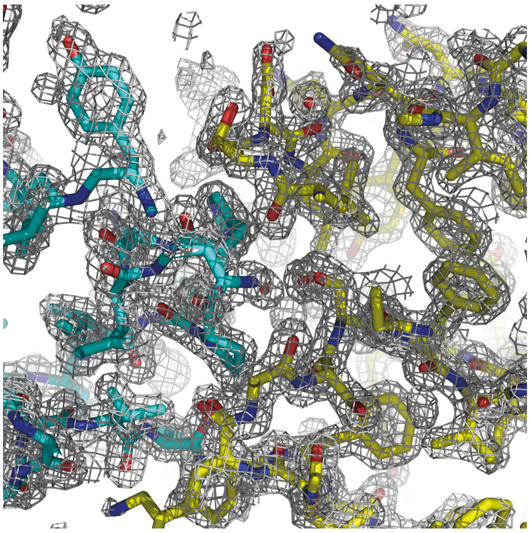

(c)

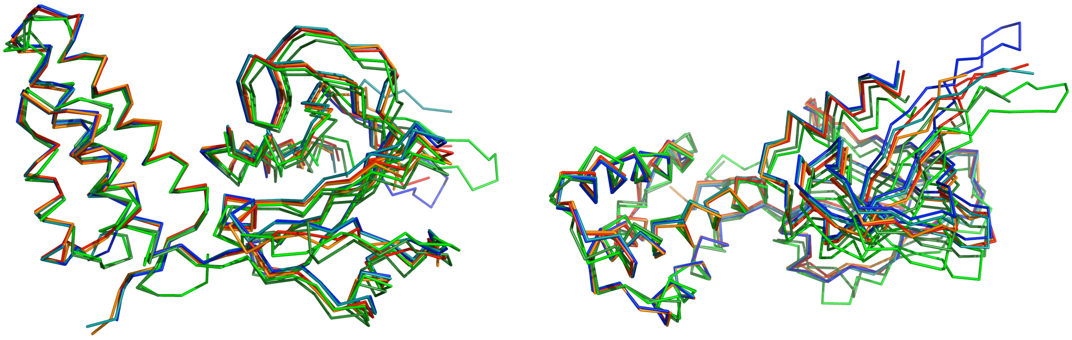

Figure S2 (a) Overlay of the two FusB molecules in the $P2_12_12$ structure (blue, light blue) and the two FusB molecules in the P1 structure (red, orange) superimposed based on domain I. Right view is seen from top of left view, 90° rotation. (b) Interface region between domains I and II. Domain I is shown in blue, domain II in yellow. The final $2F_o$-F_c map is contoured at 1.5 sigma. (c) Comparison of FusB and FusC crystal structures. The two FusC molecules in pdb 2yb5 [1] (green) were superimposed on the structures in (a) based on domain I. Compared to FusB, in FusC H2 is three residues longer and H3 and H5 start three residues later. Right view is seen from top of left view, 90° rotation.

(a) (b)

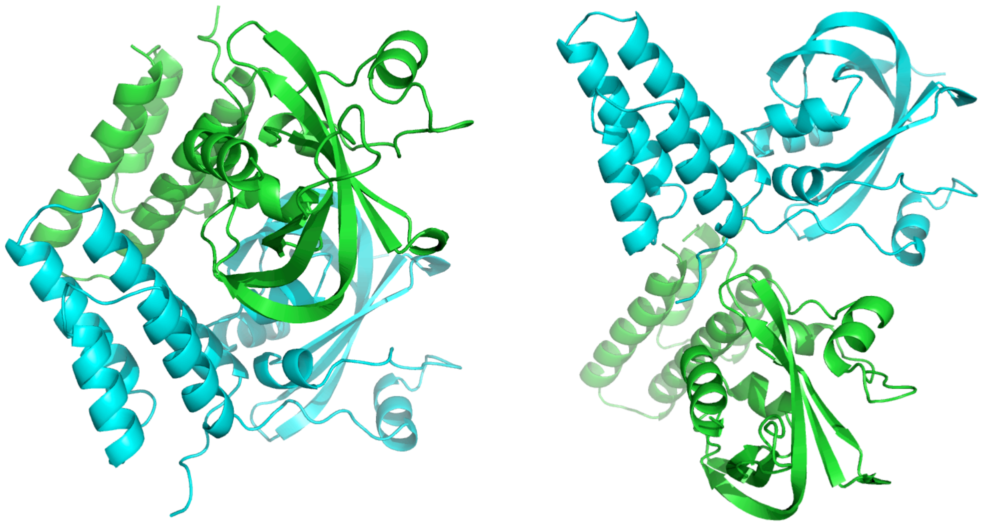

Figure S3. FusB dimers formed by crystal packing of the $P2_12_12$ crystal form. (a) “Hugging” dimer contact involving 1075 Å^2 hidden surface area. (b) “Back-to-back” dimer contacts involving 931 Å^2 hidden surface area. Similar contacts are formed in both FusB crystal forms.

Supplementary references

1. Cox, G., et al. Ribosome clearance by FusB-type proteins mediates resistance to the antibiotic fusidic acid. *Proc Natl Acad Sci U S A* **109**, 2102-2107
